# Crystal structure of tricarbon­yltris(pyri­dine-κ*N*)rhenium(I) tetra­fluorido­borate

**DOI:** 10.1107/S2056989015006180

**Published:** 2015-04-11

**Authors:** Adebomi A. Ikotun, Micheal P. Coogan, Abimbola A. Owoseni, Nattamai Bhuvanesh, Gabriel O. Egharevba

**Affiliations:** a Department of Chemistry and Industrial Chemistry, Bowen University, Iwo, Osun State, Nigeria; b Department of Chemistry, Cardiff University, Wales; c Department of Chemistry, Lancaster University, Bailrigg, England; d Department of Biological Sciences, Bowen University, Iwo, Osun State, Nigeria; e Department of Chemistry, Texas A & M University, Texas, USA; f Department of Chemistry, Obafemi Awolowo University, Ile-Ife, Nigeria

**Keywords:** crystal structure, rhenium(I) tricarbonyl complexes, tricarbonyl tris-pyridyl rhenium(I) cation, luminescent agent.

## Abstract

In the title compound, [Re(C_6_H_5_N)_3_(CO)_3_]BF_4_, the Re^I^ ion is six-coordinated by three pyridine N atoms and three carbonyl C atoms. In each case, the carbonyl C atom lies *trans* to a pyridine N atom. In the crystal, the ions are linked *via* C—H⋯F hydrogen bonds and C—H⋯π inter­actions, forming a three-dimensional framework. The F atoms of the BF_4_ anion are disordered over two positions and gave a final refined occupancy ratio of 0.705 (11):0.295 (11).

## Related literature   

For background to rhenium tricarbonyl complexes, see: Amoroso *et al.* (2008 ▸); Coogan *et al.* (2009 ▸). For the structure of tricarbonyl tris-pyridyl rhenium(I) hexa­fluoro­phosphate, see: Franklin *et al.* (2008 ▸). For the preparation of [Re(C_14_H_10_N_2_O)(CO)_3_Br] used in the synthesis, see: Al Subari *et al.* (2010 ▸); Coogan *et al.* (2009 ▸).
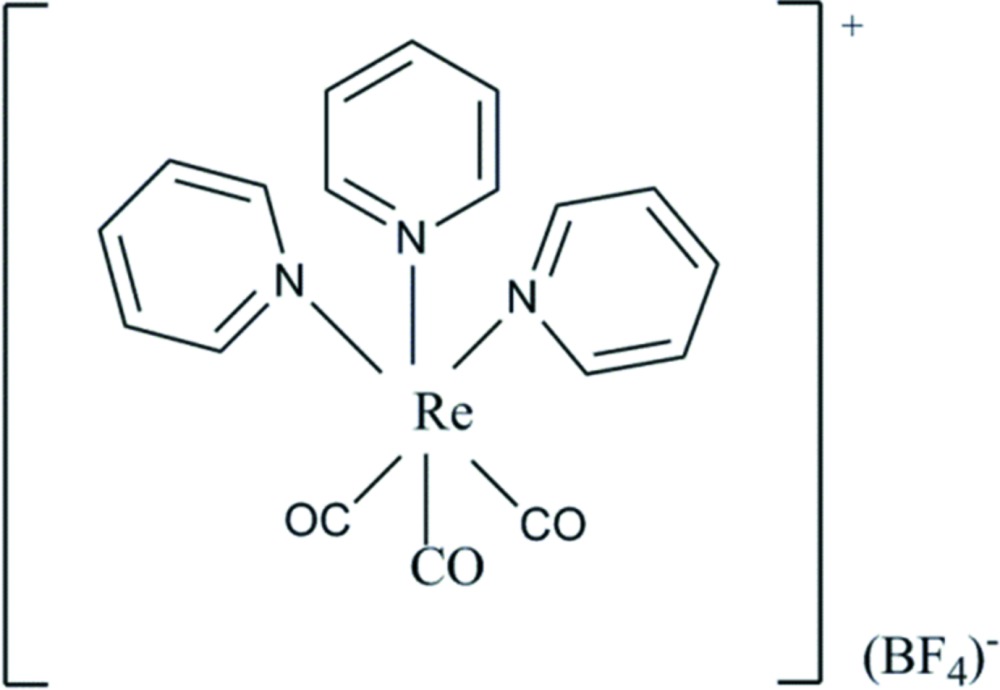


## Experimental   

### Crystal data  

[Re(C_6_H_5_N)_3_(CO)_3_]BF_4_
*M* *_r_* = 594.34
Monoclinic, 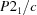

*a* = 8.1272 (12) Å
*b* = 18.718 (3) Å
*c* = 13.046 (2) Å
β = 97.317 (9)°
*V* = 1968.5 (5) Å^3^
*Z* = 4
Cu *K*α radiation
μ = 12.66 mm^−1^
*T* = 110 K
0.08 × 0.06 × 0.02 mm

### Data collection  

Bruker GADDS D8 Discover diffractometer
Absorption correction: multi-scan (*SADABS*; Sheldrick, 2006 ▸) *T*_min_ = 0.431, *T*_max_ = 0.786
39315 measured reflections
2891 independent reflections
2589 reflections with *I* > 2σ(*I*)
*R*_int_ = 0.052
θ_max_ = 60.0°
Standard reflections: 0

### Refinement  

*R*[*F* ^2^ > 2σ(*F* ^2^)] = 0.023
*wR*(*F* ^2^) = 0.048
*S* = 1.11
2891 reflections
308 parameters
172 restraints
H-atom parameters constrained
Δρ_max_ = 0.98 e Å^−3^
Δρ_min_ = −0.98 e Å^−3^

### 

Data collection: *APEX2* and *FRAMBO* (Bruker, 2004 ▸); cell refinement: *SAINT* (Bruker, 2004 ▸); data reduction: *SAINT*; program(s) used to solve structure: *SHELXS97* (Sheldrick, 2008 ▸); program(s) used to refine structure: *SHELXL97* (Sheldrick, 2008 ▸); molecular graphics: *X-SEED* (Barbour, 2001 ▸); software used to prepare material for publication: *SHELXTL* (Sheldrick, 2008 ▸) and *PLATON* (Spek, 2009 ▸).

## Supplementary Material

Crystal structure: contains datablock(s) I. DOI: 10.1107/S2056989015006180/su5093sup1.cif

Structure factors: contains datablock(s) I. DOI: 10.1107/S2056989015006180/su5093Isup2.hkl

**Fig. 1.**
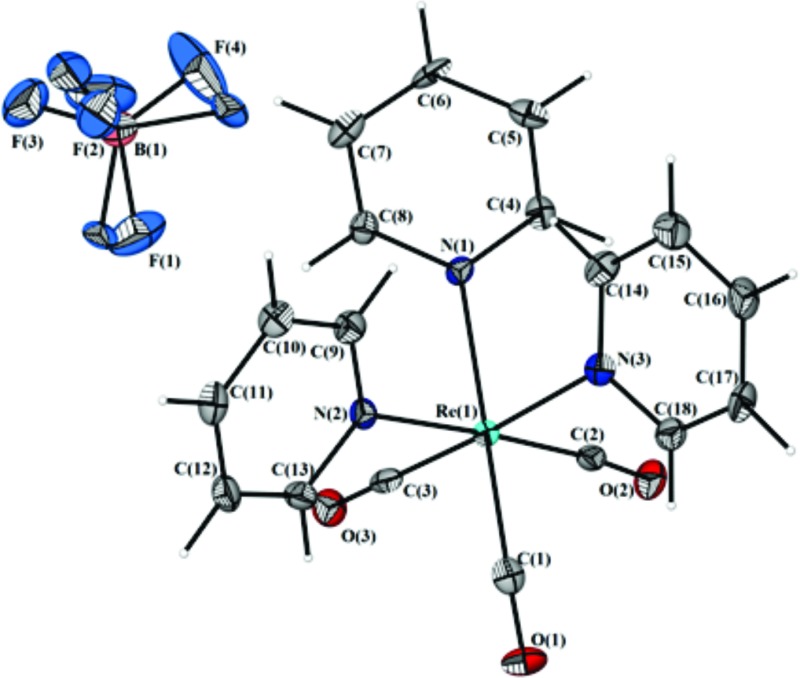
The molecular structure of the title compound, with atom labelling. Displacement ellipsoids are drawn at the 50% probability level.

. DOI: 10.1107/S2056989015006180/su5093fig1.tif
The mol­ecular structure of the title compound, with atom labelling. Displacement ellipsoids are drawn at the 50% probability level.

**Fig. 2.**
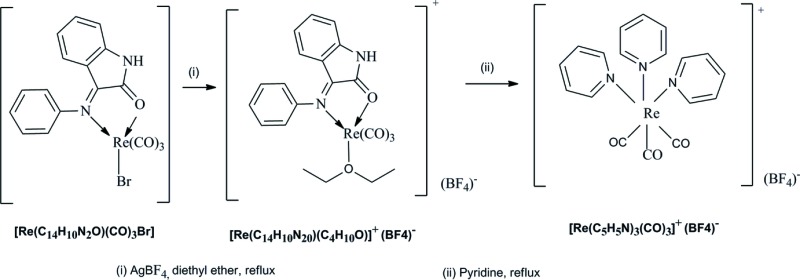
Preparation of the title compound.

. DOI: 10.1107/S2056989015006180/su5093fig2.tif
Preparation of the title compound.

CCDC reference: 1022851

Additional supporting information:  crystallographic information; 3D view; checkCIF report

## Figures and Tables

**Table 1:** Selected bond lengths ()

Re1C3	1.916(5)
Re1C1	1.924(5)
Re1C2	1.926(5)
Re1N1	2.215(3)
Re1N2	2.229(4)
Re1N3	2.240(4)

**Table 2:** Hydrogen-bond geometry (, ) *Cg*1 is the centroid of the N1/C4C8 pyrdine ring.

*D*H*A*	*D*H	H*A*	*D* *A*	*D*H*A*
C4H4*A*F3^i^	0.95	2.31	3.240(7)	165
C13H13*A*F4^ii^	0.95	2.31	3.219(12)	160
C17H17*A*F3^iii^	0.95	2.32	3.123(7)	142
C10H10*A* *Cg*1^iv^	0.95	2.61	3.302(5)	130

Symmetry codes: (i) 
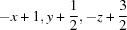
; (ii) 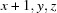; (iii) 
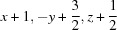
; (iv) 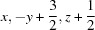.

## supplementary crystallographic information

### S1. Comment

Amoroso and coworkers (Amoroso *et al.*, 2008) prepared a novel 3-chloromethylpyridyl bipyridine tricarbonyl rhenium complex and demonstrated the suitability of this complex in Mitochondria. That report represents the first application of a luminescent agent for specific targeting of a biological entity in imaging. Recently, Coogan and co-workers (Coogan *et al.*, 2009) have also directed their research focus towards such complexes, thus preparing more novel rhenium tricarbonyl compounds to prove that heavy metals are not only erroneously termed as poisons, but can also be useful towards preparing drugs of great biological significance to man. Thus the design, syntheses and characterization of rhenium(I) tricarbonyl complexes has being of great interest due to their biological significance. The first report of the tricarbonyl tris-pyridyl rhenium(I) cation was published by Franklin *et al.* (2008), viz. tricarbonyl tris-pyridyl rhenium(I) hexafluorophosphate, which is quite similar to the title compound with some slight differences.

The molecular structure of the title complex is illustrated in Fig. 1. The Re^I^ ion is six-coordinated by three pyridine N atoms and three carbonyl C atoms.

In the crystal, the ions are linked via C-H···F hydrogen bonds and C-H···π interactions forming a three-dimensional framework (Table 1).

### S2. Experimental

The preparation of the title compound is illustrated in Fig. 2. [Re(C_14_H_10_N_2_O)(CO)_3_Br] (0.16 g, 0.28 mmol), prepared according to literature procedures (Al Subari *et al.*, 2010; Coogan *et al.*, 2009), was reacted with AgBF_4_ (0.05 g, 0.28 mmol) in 11 ml diethyl ether under nitrogen with refluxing for 35 min. The solution was then filtered through celite and to the clear filtrate pyridine (0.023 ml, 0.28 mmol) was added. The mixture was stirred for ca. 24 h. After it was poured into a vial and petroleum ether was added drop wise in excess to precipitate out the complex. This was covered with perforated foil and left overnight in the hood. Colourless block-like crystals grew on the sides of the vial.

### S3. Refinement

C-bound H atoms were placed in idealized positions and refined using a riding model: C-H = 0.95 Å with U_iso_(H) = 1.2U_eq_(C). The F atoms of the BF_4_ showed significant elongation in the thermal ellipsoids suggesting disorder over two positions; final refined occupancy ratio = 0.705 (11):0.295 (11).

### Crystal data

**Table:**

[Re(C_6_H_5_N)_3_(CO)_3_]BF_4_	*F*(000) = 1136
*M**_r_* = 594.34	*D*_x_ = 2.005 Mg m^−^^3^
Monoclinic, *P*2_1_/*c*	Cu *K*α radiation, λ = 1.54178 Å
Hall symbol: P 2ybc	Cell parameters from 2921 reflections
*a* = 8.1272 (12) Å	θ = 4.2–62.4°
*b* = 18.718 (3) Å	µ = 12.66 mm^−^^1^
*c* = 13.046 (2) Å	*T* = 110 K
β = 97.317 (9)°	Block, colourless
*V* = 1968.5 (5) Å^3^	0.08 × 0.06 × 0.02 mm
*Z* = 4	

### Data collection

**Table:**

Bruker GADDS D8 Discover diffractometer	2891 independent reflections
Radiation source: fine-focus sealed tube	2589 reflections with *I* > 2σ(*I*)
Graphite monochromator	*R*_int_ = 0.052
phi and ω scans	θ_max_ = 60.0°, θ_min_ = 4.2°
Absorption correction: multi-scan (*SADABS*; Sheldrick, 2006)	*h* = −9→9
*T*_min_ = 0.431, *T*_max_ = 0.786	*k* = −21→21
39315 measured reflections	*l* = −14→14

### Refinement

**Table:**

Refinement on *F*^2^	Primary atom site location: structure-invariant direct methods
Least-squares matrix: full	Secondary atom site location: difference Fourier map
*R*[*F*^2^ > 2σ(*F*^2^)] = 0.023	Hydrogen site location: inferred from neighbouring sites
*wR*(*F*^2^) = 0.048	H-atom parameters constrained
*S* = 1.11	*w* = 1/[σ^2^(*F*_o_^2^) + (0.0131*P*)^2^ + 6.6179*P*] where *P* = (*F*_o_^2^ + 2*F*_c_^2^)/3
2891 reflections	(Δ/σ)_max_ = 0.001
308 parameters	Δρ_max_ = 0.98 e Å^−^^3^
172 restraints	Δρ_min_ = −0.98 e Å^−^^3^

### Special details

**Table:**

Geometry. All e.s.d.'s (except the e.s.d. in the dihedral angle between two l.s. planes) are estimated using the full covariance matrix. The cell e.s.d.'s are taken into account individually in the estimation of e.s.d.'s in distances, angles and torsion angles; correlations between e.s.d.'s in cell parameters are only used when they are defined by crystal symmetry. An approximate (isotropic) treatment of cell e.s.d.'s is used for estimating e.s.d.'s involving l.s. planes.
Refinement. Refinement of *F*^2^ against ALL reflections. The weighted *R*-factor *wR* and goodness of fit *S* are based on *F*^2^, conventional *R*-factors *R* are based on *F*, with *F* set to zero for negative *F*^2^. The threshold expression of *F*^2^ > σ(*F*^2^) is used only for calculating *R*-factors(gt) *etc*. and is not relevant to the choice of reflections for refinement. *R*-factors based on *F*^2^ are statistically about twice as large as those based on *F*, and *R*- factors based on ALL data will be even larger.

### Fractional atomic coordinates and isotropic or equivalent isotropic displacement parameters (Å^2^)

**Table:**

	*x*	*y*	*z*	*U*_iso_*/*U*_eq_	Occ. (<1)
Re1	0.90696 (2)	0.817237 (10)	0.800171 (15)	0.01465 (7)	
C1	1.1344 (6)	0.8090 (2)	0.8614 (3)	0.0212 (10)	
C2	0.9740 (5)	0.9040 (2)	0.7377 (3)	0.0188 (10)	
C3	0.9626 (5)	0.7679 (2)	0.6806 (4)	0.0187 (10)	
C4	0.5751 (6)	0.8920 (2)	0.7123 (3)	0.0209 (10)	
H4A	0.6364	0.9327	0.7387	0.025*	
C5	0.4181 (6)	0.9021 (3)	0.6613 (4)	0.0235 (10)	
H5A	0.3714	0.9486	0.6534	0.028*	
C6	0.3314 (6)	0.8441 (3)	0.6224 (4)	0.0253 (10)	
H6A	0.2237	0.8500	0.5857	0.030*	
C7	0.3993 (6)	0.7762 (3)	0.6360 (4)	0.0255 (11)	
H7A	0.3388	0.7352	0.6100	0.031*	
C8	0.5569 (5)	0.7699 (2)	0.6884 (3)	0.0190 (10)	
H8A	0.6045	0.7236	0.6978	0.023*	
C9	0.7018 (6)	0.7040 (2)	0.9139 (3)	0.0223 (11)	
H9A	0.6199	0.7405	0.9073	0.027*	
C10	0.6690 (6)	0.6415 (2)	0.9644 (3)	0.0226 (11)	
H10A	0.5663	0.6355	0.9910	0.027*	
C11	0.7859 (6)	0.5882 (2)	0.9758 (4)	0.0255 (11)	
H11A	0.7674	0.5452	1.0112	0.031*	
C12	0.9321 (6)	0.5994 (2)	0.9336 (4)	0.0246 (11)	
H12A	1.0154	0.5635	0.9392	0.030*	
C13	0.9560 (6)	0.6625 (2)	0.8837 (4)	0.0221 (11)	
H13A	1.0565	0.6691	0.8548	0.027*	
C14	0.6690 (6)	0.8790 (2)	0.9558 (4)	0.0233 (11)	
H14A	0.5847	0.8586	0.9074	0.028*	
C15	0.6234 (6)	0.9127 (3)	1.0420 (4)	0.0289 (12)	
H15A	0.5100	0.9150	1.0526	0.035*	
C16	0.7436 (6)	0.9430 (3)	1.1123 (4)	0.0295 (12)	
H16A	0.7151	0.9666	1.1720	0.035*	
C17	0.9070 (6)	0.9383 (2)	1.0939 (4)	0.0264 (11)	
H17A	0.9930	0.9586	1.1412	0.032*	
C18	0.9439 (6)	0.9040 (2)	1.0070 (4)	0.0217 (10)	
H18A	1.0567	0.9014	0.9952	0.026*	
N1	0.6466 (4)	0.82675 (18)	0.7269 (3)	0.0160 (8)	
N2	0.8427 (4)	0.71536 (18)	0.8741 (3)	0.0173 (8)	
N3	0.8279 (4)	0.87363 (19)	0.9373 (3)	0.0179 (8)	
O1	1.2715 (4)	0.80628 (18)	0.8956 (3)	0.0309 (8)	
O2	1.0248 (4)	0.95285 (17)	0.6991 (2)	0.0273 (8)	
O3	0.9949 (4)	0.74082 (17)	0.6064 (2)	0.0255 (7)	
B1	0.3395 (7)	0.5865 (3)	0.7653 (5)	0.0338 (14)	0.705 (11)
F1	0.5081 (9)	0.5915 (5)	0.7637 (9)	0.056 (3)	0.705 (11)
F2	0.3088 (9)	0.5538 (3)	0.8585 (5)	0.0468 (17)	0.705 (11)
F3	0.2785 (6)	0.5397 (3)	0.6835 (4)	0.0522 (18)	0.705 (11)
F4	0.2648 (13)	0.6503 (3)	0.7527 (8)	0.082 (3)	0.705 (11)
B1A	0.3395 (7)	0.5865 (3)	0.7653 (5)	0.0338 (14)	0.295 (11)
F1A	0.5017 (19)	0.5630 (8)	0.776 (2)	0.026 (4)	0.295 (11)
F2A	0.236 (2)	0.5462 (8)	0.8115 (18)	0.061 (5)	0.295 (11)
F3A	0.2854 (15)	0.5990 (11)	0.6583 (9)	0.069 (6)	0.295 (11)
F4A	0.3349 (16)	0.6580 (6)	0.8070 (12)	0.035 (3)	0.295 (11)

### Atomic displacement parameters (Å^2^)

**Table:**

	*U*^11^	*U*^22^	*U*^33^	*U*^12^	*U*^13^	*U*^23^
Re1	0.01373 (11)	0.01449 (11)	0.01556 (11)	−0.00004 (9)	0.00123 (7)	−0.00028 (9)
C1	0.029 (3)	0.022 (2)	0.014 (2)	0.001 (2)	0.004 (2)	0.000 (2)
C2	0.014 (2)	0.022 (2)	0.019 (2)	0.003 (2)	−0.003 (2)	−0.007 (2)
C3	0.012 (2)	0.017 (2)	0.026 (3)	−0.0014 (18)	−0.001 (2)	0.008 (2)
C4	0.024 (3)	0.018 (2)	0.021 (3)	0.000 (2)	0.004 (2)	0.000 (2)
C5	0.020 (2)	0.028 (2)	0.024 (2)	0.0068 (19)	0.007 (2)	0.005 (2)
C6	0.015 (2)	0.032 (2)	0.027 (2)	−0.0113 (18)	−0.0049 (19)	0.005 (2)
C7	0.025 (3)	0.029 (2)	0.022 (2)	−0.010 (2)	−0.001 (2)	0.003 (2)
C8	0.022 (3)	0.017 (2)	0.017 (2)	−0.0016 (19)	0.001 (2)	0.000 (2)
C9	0.022 (3)	0.023 (2)	0.021 (3)	−0.001 (2)	0.001 (2)	−0.002 (2)
C10	0.026 (3)	0.023 (3)	0.020 (3)	−0.005 (2)	0.006 (2)	0.006 (2)
C11	0.037 (3)	0.019 (2)	0.021 (3)	−0.005 (2)	0.002 (2)	0.000 (2)
C12	0.029 (3)	0.016 (2)	0.027 (3)	0.004 (2)	−0.001 (2)	−0.002 (2)
C13	0.017 (2)	0.025 (3)	0.024 (3)	0.003 (2)	0.003 (2)	−0.004 (2)
C14	0.023 (3)	0.027 (3)	0.020 (3)	−0.005 (2)	0.002 (2)	0.000 (2)
C15	0.030 (3)	0.030 (3)	0.031 (3)	−0.001 (2)	0.015 (2)	−0.004 (2)
C16	0.036 (3)	0.027 (3)	0.028 (3)	−0.003 (2)	0.013 (2)	−0.012 (2)
C17	0.031 (3)	0.023 (3)	0.025 (3)	−0.008 (2)	0.000 (2)	−0.008 (2)
C18	0.021 (3)	0.018 (2)	0.025 (3)	−0.001 (2)	0.001 (2)	0.003 (2)
N1	0.0159 (19)	0.0144 (19)	0.0174 (19)	−0.0027 (15)	0.0015 (15)	0.0026 (16)
N2	0.020 (2)	0.0159 (18)	0.0159 (19)	0.0004 (16)	0.0010 (16)	−0.0024 (16)
N3	0.021 (2)	0.0170 (19)	0.015 (2)	−0.0008 (16)	0.0030 (17)	0.0034 (16)
O1	0.0159 (19)	0.042 (2)	0.033 (2)	0.0020 (15)	−0.0057 (16)	0.0022 (17)
O2	0.0305 (19)	0.0204 (17)	0.0315 (19)	−0.0066 (15)	0.0058 (16)	0.0017 (16)
O3	0.0288 (19)	0.0264 (18)	0.0222 (18)	−0.0004 (15)	0.0060 (15)	−0.0044 (16)
B1	0.025 (3)	0.031 (3)	0.048 (4)	−0.004 (3)	0.013 (3)	−0.005 (3)
F1	0.034 (3)	0.092 (8)	0.045 (5)	−0.021 (4)	0.014 (3)	−0.029 (6)
F2	0.045 (4)	0.035 (3)	0.063 (4)	−0.007 (3)	0.016 (3)	0.006 (3)
F3	0.044 (3)	0.043 (4)	0.062 (3)	−0.008 (2)	−0.019 (2)	0.001 (3)
F4	0.121 (7)	0.036 (3)	0.106 (7)	0.031 (4)	0.075 (6)	0.029 (4)
B1A	0.025 (3)	0.031 (3)	0.048 (4)	−0.004 (3)	0.013 (3)	−0.005 (3)
F1A	0.017 (5)	0.023 (8)	0.043 (8)	0.003 (5)	0.017 (5)	−0.001 (7)
F2A	0.031 (8)	0.032 (6)	0.130 (15)	−0.009 (6)	0.044 (9)	−0.002 (9)
F3A	0.038 (7)	0.111 (16)	0.054 (6)	0.018 (7)	−0.010 (5)	−0.010 (7)
F4A	0.028 (7)	0.017 (5)	0.059 (9)	0.009 (4)	0.001 (6)	0.009 (5)

### Geometric parameters (Å, º)

**Table:**

Re1—C3	1.916 (5)	C10—C11	1.372 (7)
Re1—C1	1.924 (5)	C10—H10A	0.9500
Re1—C2	1.926 (5)	C11—C12	1.387 (7)
Re1—N1	2.215 (3)	C11—H11A	0.9500
Re1—N2	2.229 (4)	C12—C13	1.376 (6)
Re1—N3	2.240 (4)	C12—H12A	0.9500
C1—O1	1.148 (5)	C13—N2	1.346 (6)
C2—O2	1.146 (5)	C13—H13A	0.9500
C3—O3	1.152 (5)	C14—N3	1.348 (6)
C4—N1	1.355 (6)	C14—C15	1.381 (7)
C4—C5	1.375 (6)	C14—H14A	0.9500
C4—H4A	0.9500	C15—C16	1.375 (7)
C5—C6	1.356 (7)	C15—H15A	0.9500
C5—H5A	0.9500	C16—C17	1.382 (7)
C6—C7	1.389 (7)	C16—H16A	0.9500
C6—H6A	0.9500	C17—C18	1.369 (7)
C7—C8	1.378 (6)	C17—H17A	0.9500
C7—H7A	0.9500	C18—N3	1.349 (6)
C8—N1	1.350 (5)	C18—H18A	0.9500
C8—H8A	0.9500	B1—F4	1.340 (8)
C9—N2	1.334 (6)	B1—F1	1.376 (9)
C9—C10	1.385 (6)	B1—F2	1.412 (8)
C9—H9A	0.9500	B1—F3	1.420 (7)
			
C3—Re1—C1	89.17 (18)	C10—C11—C12	117.7 (4)
C3—Re1—C2	87.34 (18)	C10—C11—H11A	121.2
C1—Re1—C2	86.22 (18)	C12—C11—H11A	121.2
C3—Re1—N1	89.95 (16)	C13—C12—C11	119.8 (4)
C1—Re1—N1	178.97 (16)	C13—C12—H12A	120.1
C2—Re1—N1	93.21 (15)	C11—C12—H12A	120.1
C3—Re1—N2	91.85 (15)	N2—C13—C12	122.6 (4)
C1—Re1—N2	91.02 (16)	N2—C13—H13A	118.7
C2—Re1—N2	177.13 (16)	C12—C13—H13A	118.7
N1—Re1—N2	89.54 (13)	N3—C14—C15	122.9 (4)
C3—Re1—N3	176.99 (16)	N3—C14—H14A	118.6
C1—Re1—N3	93.69 (16)	C15—C14—H14A	118.6
C2—Re1—N3	93.77 (16)	C16—C15—C14	119.3 (5)
N1—Re1—N3	87.20 (13)	C16—C15—H15A	120.3
N2—Re1—N3	87.18 (12)	C14—C15—H15A	120.3
O1—C1—Re1	177.4 (4)	C15—C16—C17	118.4 (4)
O2—C2—Re1	174.7 (4)	C15—C16—H16A	120.8
O3—C3—Re1	177.1 (4)	C17—C16—H16A	120.8
N1—C4—C5	123.2 (4)	C18—C17—C16	119.3 (4)
N1—C4—H4A	118.4	C18—C17—H17A	120.3
C5—C4—H4A	118.4	C16—C17—H17A	120.3
C6—C5—C4	118.4 (4)	N3—C18—C17	123.3 (4)
C6—C5—H5A	120.8	N3—C18—H18A	118.4
C4—C5—H5A	120.8	C17—C18—H18A	118.4
C5—C6—C7	120.4 (4)	C8—N1—C4	117.1 (4)
C5—C6—H6A	119.8	C8—N1—Re1	122.6 (3)
C7—C6—H6A	119.8	C4—N1—Re1	120.1 (3)
C8—C7—C6	118.1 (4)	C9—N2—C13	117.2 (4)
C8—C7—H7A	121.0	C9—N2—Re1	124.4 (3)
C6—C7—H7A	121.0	C13—N2—Re1	118.3 (3)
N1—C8—C7	122.8 (4)	C14—N3—C18	116.8 (4)
N1—C8—H8A	118.6	C14—N3—Re1	123.9 (3)
C7—C8—H8A	118.6	C18—N3—Re1	119.4 (3)
N2—C9—C10	123.2 (4)	F4—B1—F1	112.0 (6)
N2—C9—H9A	118.4	F4—B1—F2	111.4 (6)
C10—C9—H9A	118.4	F1—B1—F2	109.1 (7)
C11—C10—C9	119.5 (4)	F4—B1—F3	110.4 (7)
C11—C10—H10A	120.3	F1—B1—F3	106.5 (6)
C9—C10—H10A	120.3	F2—B1—F3	107.2 (5)

### Hydrogen-bond geometry (Å, º)

Cg1 is the centroid of the N1/C4–C8 pyrdine ring.

**Table:**

*D*—H···*A*	*D*—H	H···*A*	*D*···*A*	*D*—H···*A*
C4—H4*A*···F3^i^	0.95	2.31	3.240 (7)	165
C13—H13*A*···F4^ii^	0.95	2.31	3.219 (12)	160
C17—H17*A*···F3^iii^	0.95	2.32	3.123 (7)	142
C10—H10*A*···*Cg*1^iv^	0.95	2.61	3.302 (5)	130

Symmetry codes: (i) −*x*+1, *y*+1/2, −*z*+3/2; (ii) *x*+1, *y*, *z*; (iii) *x*+1, −*y*+3/2, *z*+1/2; (iv) *x*, −*y*+3/2, *z*+1/2.
