# Supplementary figures and images for: Crystal structure of tricarbon­yltris(pyri­dine-κN)rhenium(I) tetra­fluorido­borate
Source: Acta Crystallogr E Crystallogr Commun. 2015 Apr 11;71(Pt 5):m106–7. doi: 10.1107/S2056989015006180 (PMC4420038; doi:10.1107/S2056989015006180)

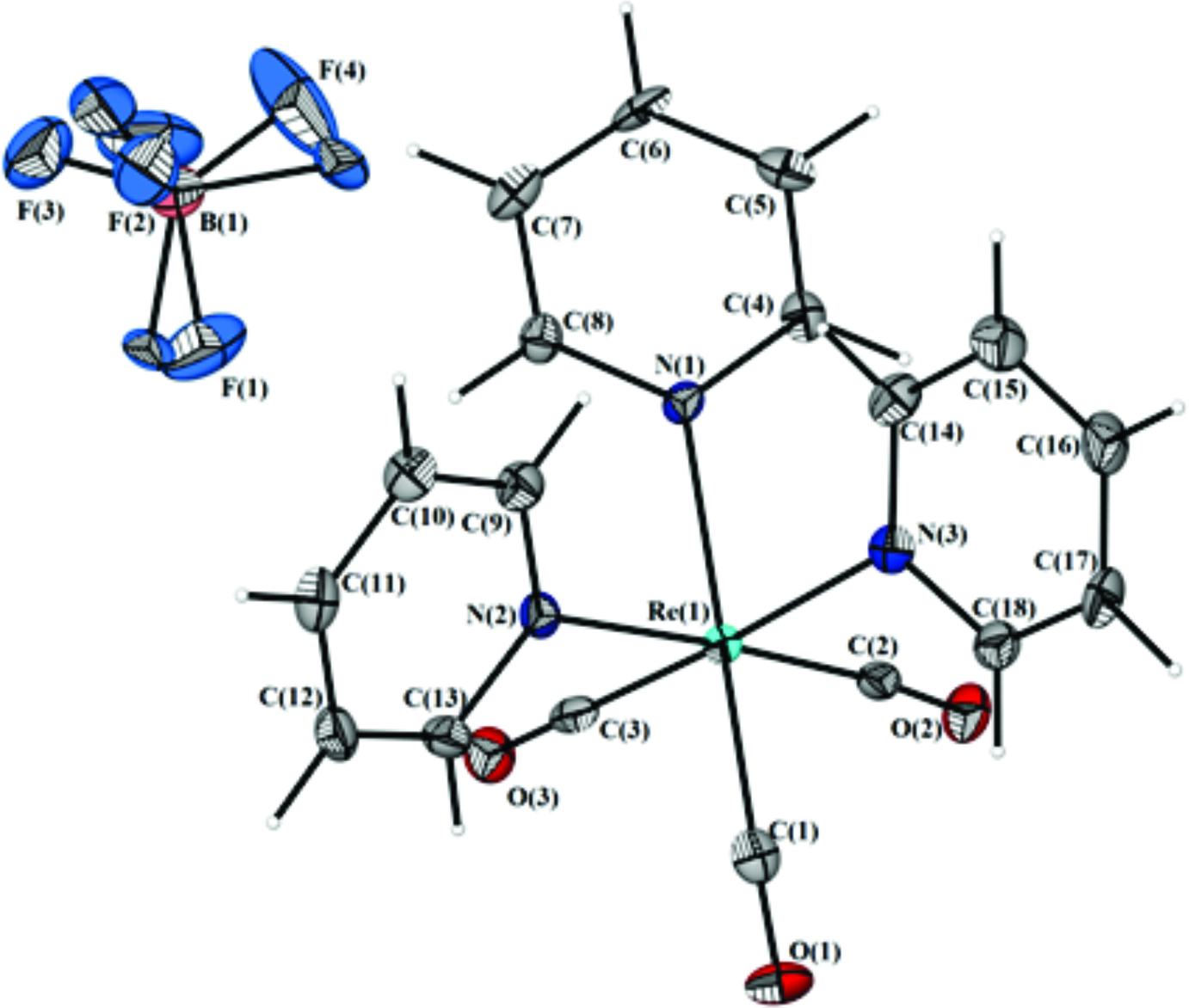

Supplement: Supplementary file 3 [file e-71-0m106-fig1.tif]

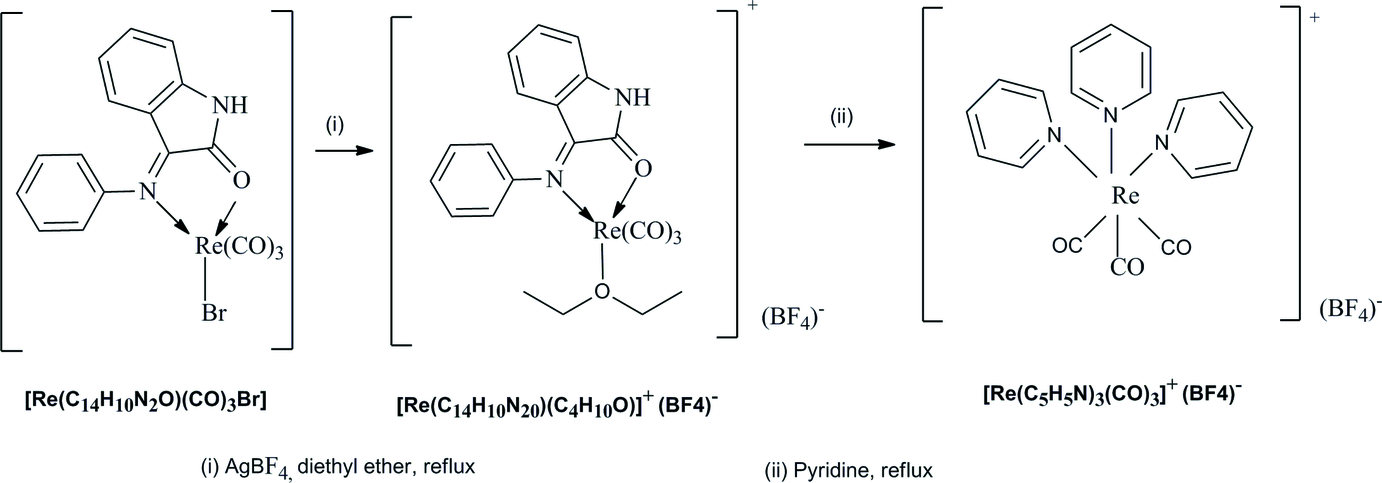

Supplement: Supplementary file 4 [file e-71-0m106-fig2.tif]
